# Supplementary material for: Actin and an unconventional myosin motor, TgMyoF, control the organization and dynamics of the endomembrane network in Toxoplasma gondii
Source: PLoS Pathog. 2021 Feb 2;17(2):e1008787. doi: 10.1371/journal.ppat.1008787 (PMC7880465; doi:10.1371/journal.ppat.1008787)
Supplement: S1 Table — * indicates number of vacuoles instead of number of parasites. (DOCX) [file ppat.1008787.s013.docx]

| **Vesicle** | **Treatment** | **Parasite Line** | **N**  **(parasites)** | **N**  **(vesicles)** | **Run Frequency (± SEM)** | **Velocity (µm/s)**  **(± SEM)** |
| --- | --- | --- | --- | --- | --- | --- |
| Rab6 | DMSO | RH | 89 | 651 | 5.8 ± 0.44 | 0.92 ± 0.1 |
| Rab6 | CytoD | RH | 79 | 19 | 0.24 ± 0.05 | N/A |
| Rab6 | Oryzalin | RH | 32* | 354 | 11.71 ± 0.25 | 0.54 ± 0.09 |
| Rab6 | Ethanol | TgMyoF-mAID | 76 | 554 | 7.6 ± 0.7 | 0.91 ± 0.1 |
| Rab6 | IAA | TgMyoF-mAID | 50 | 151 | 3.0 ± 0.45 | 0.94 ± 0.05 |
| Rop1 | Ethanol | TgMyoF-mAID | 44 | 528 | 11.1 ± 1.1 | 1.34 ± 0.02 |
| Rop1 | IAA | TgMyoF-mAID | 57 | 110 | 1.4 ± 0.24 | 1.25 ± 0.05 |
