# Supplementary material for: Actin and an unconventional myosin motor, TgMyoF, control the organization and dynamics of the endomembrane network in Toxoplasma gondii
Source: PLoS Pathog. 2021 Feb 2;17(2):e1008787. doi: 10.1371/journal.ppat.1008787 (PMC7880465; doi:10.1371/journal.ppat.1008787)
Supplement: S2 Table — (DOCX) [file ppat.1008787.s014.docx]

| **Plasmid Name** | **Purpose** | **Reference** |
| --- | --- | --- |
| pTKOII_MyoF_mAID | Endogenous tagging of TgMyoF genomic locus | This study |
| pmin-EmGFP-Rab6-ble | Ectopic expression of TgRab6 | This study |
| pmin-NeonGreen-Rab5 | Pmin-NeonGreen-Rab7 | This study |
| pTg-HA-Rab5 | Subcloning | Robibaro B, Stedman TT, Coppens I, Ngô HM, Pypaert M, Bivona T, et al. Toxoplasma gondii Rab5 enhances cholesterol acquisition from host cells. Cell Microbiol. 2002; |
| pTg-HA-Rab7 | Subcloning | Parussini F, Coppens I, Shah PP, Diamond SL, Carruthers VB. Cathepsin L occupies a vacuolar compartment and is a protein maturase within the endo/exocytic system of Toxoplasma gondii. Mol Microbiol. 2010;76(6):1340–57 |
| ptub-SAG1-ΔGPI-HDEL | Fluorescent labeling of ER | This study |
| ptub-SAG1-ΔGPI | Fluorescent labeling of Dense Granules | Heaslip AT, Nelson SR, Warshaw DM. Dense granule trafficking in Toxoplasma gondii requires a unique class 27 myosin and actin filaments. Mol Biol Cell. 2016;27(13):2080–9. |
| ptub-Rop1-NeonGreenFP | Ectopic expression of Rop1 | This study & Striepen B, Soldati D, Garcia-Reguet N, Dubremetz JF, Roos DS. Targeting of soluble proteins to the rhoptries and micronemes in Toxoplasma gondii. Mol Biochem Parasitol. 2001;113(1):45–53. |
| GFP-Syntaxin6 | Ectopic expression of syntaxin6 | Jackson AJ, Clucas C, Mamczur NJ, Ferguson DJ, Meissner M. Toxoplasma gondii Syntaxin 6 Is Required for Vesicular Transport Between Endosomal-Like Compartments and the Golgi Complex. Traffic. 2013;14(11):1166–81. |
| GFP-DrpB | Ectopic expression of DrpB | Breinich MS, Ferguson DJ, Foth BJ, van Dooren GG, Lebrun M, Quon D V, et al. A dynamin is required for the biogenesis of secretory organelles in Toxoplasma gondii. Curr Biol. 2009;19(4):277–86 |
| Ty1-NeonGreen Pave | Subcloning | Kind gift from Dr. Chris de Graffienried (Brown University, Providence RI) |
| ptub-Grasp55-GFP/mCherry | Fluorescent labeling of the Golgi | Pelletier L, Stern CA, Pypaert M, Sheff D, Ngô HM, Roper N, et al. Golgi biogenesis in Toxoplasma gondii. Nature. 2002 Aug 1;418(6897):548–52 |
| pmin-Centrin1-GFP | Fluorescent labeling of the centrosomes | Hu K. Organizational changes of the daughter basal complex during the parasite replication of Toxoplasma gondii. PLoS Pathog. 2008;4(1):0108–21 |
