# Supplementary material for: Actin and an unconventional myosin motor, TgMyoF, control the organization and dynamics of the endomembrane network in Toxoplasma gondii
Source: PLoS Pathog. 2021 Feb 2;17(2):e1008787. doi: 10.1371/journal.ppat.1008787 (PMC7880465; doi:10.1371/journal.ppat.1008787)
Supplement: S3 Table — (DOCX) [file ppat.1008787.s015.docx]

| **Primer Name** | **Sequence** |
| --- | --- |
| AID-HA ultramer S | agatctatggtttcctgccaaaaatcaagcggtggcccggaggcggcggcgtt  cgtgaaggtatcaatggacggagcaccgtacttgaggaaaatcgatttgagga  tgtataaatacccatacgatgttccagattacgcttaacttaag |
| AID HA F | gggcgcgaggtcgagctgtcggtaccagatctatgaaggagaagagtgcttgt |
| AID HA R | gtttaaacagatcccttaagttaagcgtaatctggaacatcgtatgggta  tttatacatcctcaaatcga |
| Rab6 F | gcggaggcgacagttgcggg |
| Rab6 R | gagaggaaaacgagagacgggcagcttctgtttacttaagtcagcaagagcaggatgaag |
| EmGFP-R6 F | gtgcgtttctgctctacggggcgttgtcagatccgctagcatgggactcgtgagcaaggg |
| EmGFP-R6 R | caagtttgtatttgttcaaccccgcaactgtcgcctccgccttgtacagctcgtccatgc |
| Rab5a F | agaggtttcgaatctgctga |
| Rab5a R | gaaaagagaggaaaacgagagacgggcagcttctgtttactcaacttttgcctccacatg |
| NeonFP-R5a F | tagtagtgcgtttctgctctacggggcgttgtcagatccgatggtctcgaaaggtgagga |
| NeonFP-R5a R | ggcgagagtacgcgcggtcctcagcagattcgaaacctctcttatacaattcgtccatcc |
| Rab7 F | ccgcccaagaagaaggctct |
| Rab7 R | gagaggaaaacgagagacgggcagcttctgtttacttaagtcagcagcagccgccgctgc |
| NeonFP-R7 F | gtgcgtttctgctctacggggcgttgtcagatccgctagcatggtctcgaaaggtgagga |
| NeonFP R7 R | cgaggatgatgactttcaagagagccttcttcttgggcggcttatacaattcgtccatcc |
| Rop1 F | gttggcattttttcttgaattcccttttagatccgctagcatggagcaaaggctgccaat |
| Rop1 R | ctggcaagctggccatattgtcttcctcacctttcgagacttgcgatccatcatcctgct |
| NeonFP Rop1 F | caacgcacgagcttcatata |
| NeonFP Rop1 R | aggaaaacgagagacgggcagcttctgtttacttaagttacttatacaattcgtccatcc |
| MyoF gF1 | cgtcgtcgagtgtatctacgg |
| MyoF gF2 | actgagagtttctgtttttcct |
| MyoF gR1 | taatgtatgctatacgaagtta |
| MyoF gR2 | aagaagcactcgagtccatttc |
| Auxin R3 | gtttaaacagatcccttaagttaagcgtaatctggaacatcgtatgggtatttatacatcctcaaatcga |
| HDEL UltramerS | cggcatggacgagctgtacaaggcgcatgatgaactgtgacttaagtaaacagaagctgcccgtctc |
| HDEL Ultramer AS | gagacgggcagcttctgtttacttaagtcacagttcatcatgcgccttgtacagctc gtccatgccg |
