# Supplementary material for: Actin and an unconventional myosin motor, TgMyoF, control the organization and dynamics of the endomembrane network in Toxoplasma gondii
Source: PLoS Pathog. 2021 Feb 2;17(2):e1008787. doi: 10.1371/journal.ppat.1008787 (PMC7880465; doi:10.1371/journal.ppat.1008787)
Supplement: S4 Table — (DOCX) [file ppat.1008787.s016.docx]

| **Primary Antibody** | **Dilution** | **Reference/Source** |
| --- | --- | --- |
| Rabbit Anti-TgCPL | 1:500 | Larson ET, Parussini F, Huynh MH, Giebel JD, Kelley AM, Zhang L, et al. Toxoplasma gondii cathepsin L Is the primary target of the Invasion-inhibitory compound morpholinurea-leucylhomophenyl-vinyl sulfone phenyl. J Biol Chem. 2009; |
| Mouse Anti-IMC | 1:1000 | Dr. Gary Ward; Univ. of Vermont |
| Rat anti-HA, Clone 3F10 | 1:500 | Roche Cat #11867423001 |
| Mouse anti-AMA1 | 1:1000 | Donahue CG, Carruthers VB, Gilk SD, Ward GE. The Toxoplasma homolog of Plasmodium apical membrane antigen-1 (AMA-1) is a microneme protein secreted in response to elevated intracellular calcium levels. Mol Biochem Parasitol. 2000 |
| Rabbit anti-GAP45 | 1:1000 | Gaskins E, Gilk S, DeVore N, Mann T, Ward G, Beckers C. Identification of the membrane receptor of a class XIV myosin in Toxoplasma gondii. J Cell Biol. 2004 |
| Mouse anti-tubulin | 1:1000 | MiliporeSigma T6074 |
| Rat anti-SORTLR | 1:500 | Venugopal K, Werkmeister E, Barois N, Saliou JM, Poncet A, Huot L, et al. Dual role of the Toxoplasma gondii clathrin adaptor AP1 in the sorting of rhoptry and microneme proteins and in parasite division. Vol. 13, PLoS Pathogens. 2017. 1–38 p. |
| **Secondary Antibody** | **Dilution** | **Catalog Information** |
| Goat anti-mouse AlexaFluor 546 | 1:1000 | ThermoFisher Cat # A11030 |
| Goat anti-mouse AlexaFluor 647 | 1:1000 | ThermoFisher Cat # A21235 |
| Goat anti-Rat AlexaFluor 488 | 1:1000 | ThermoFisher Cat # A11006 |
| Goat anti-Rat AlexaFluor 546 | 1:500 | ThermoFisher Cat# A11081 |
| Goat anti-mouse HRP | 1:10,000 | ThermoFisher Cat # 31430 |
| Goat anti-rat HRP | 1:5,000 | ThermoFisher Cat # 31470 |
