# Supplementary material for: Actin and an unconventional myosin motor, TgMyoF, control the organization and dynamics of the endomembrane network in Toxoplasma gondii
Source: PLoS Pathog. 2021 Feb 2;17(2):e1008787. doi: 10.1371/journal.ppat.1008787 (PMC7880465; doi:10.1371/journal.ppat.1008787)
Supplement: S5 Table — (DOCX) [file ppat.1008787.s017.docx]

| **Gene Name** | **ToxoDB Accession number** |
| --- | --- |
| Rab6 | TgME49_310460 |
| Rab5a | TgME49_267810 |
| Rab7 | TgME49_248880 |
| DrpB | TgME49_321620 |
| Syntaxin 6 | TgME49_300240 |
| MyoF | TgME49_278870 |
| Rop1 | TgME49_309590 |
