# Supplementary material for: Actin and an unconventional myosin motor, TgMyoF, control the organization and dynamics of the endomembrane network in Toxoplasma gondii
Source: PLoS Pathog. 2021 Feb 2;17(2):e1008787. doi: 10.1371/journal.ppat.1008787 (PMC7880465; doi:10.1371/journal.ppat.1008787)
Supplement: Attachment — Submitted filename: Carmeille et al_Coverletter.docx [file ppat.1008787.s018.docx]

18^th^ December, 2020

Dear Dr. Gubbels,

We thank the reviewers for their positive comments and feedback on our paper. We have included new data in this revision to address the reviewers’ major concerns as follows:

- Visualization and quantification of Rab6 vesicle motion after microtubule depolymerization with oryzalin (Fig. 3)
- Imaging of endoplasmic reticulum movements after actin and microtubule depolymerization (Fig. 7)
- Co-localization experiments with EmGFP-Rab6 and a marker for the trans-Golgi (anti-SORTLR) in both RH parasites (Fig. 1C) and in TgMyoF-AID parasites treated with ethanol and IAA (Fig. S5)
- A time-course experiment looking at the timing of Rab6 compartment and Golgi fragmentation to discern if fragmentation of one of these compartments is a downstream consequence of the other (Fig. 8)
- Tir1 parental parasites were treated with ethanol or IAA and effects on apicoplast inheritance, Golgi fragmentation and Rab6 fragmentation were assessed (Fig. S3 and S4).

A point by point response to the reviewer’s major and minor concerns can be found below. The reviewers’ comments are in black, our response is in orange italics.

We hope that these changes meet the satisfaction of yourself and the reviewers.

Kind Regards,

Dr. Aoife Heaslip

**Part II – Major Issues: Key Experiments Required for Acceptance**
Please use this section to detail the key new experiments or modifications of existing experiments that should be absolutely required to validate study conclusions.

Generally, there should be no more than 3 such required experiments or major modifications for a "Major Revision" recommendation. If more than 3 experiments are necessary to validate the study conclusions, then you are encouraged to recommend "Reject".

Reviewer #1: Fig 3A:
Rab6 is known to interact with microtubule motors in mammalian cells. Thus, a control with a microtubule destabilizing agent should be included to exclude a role of this cytoskeleton in Rab6 vesicle motion.

*This experiment has been performed and the data added to Fig. 3 and the results described in the results section (Lines 193-206). We see a 30% decrease in both the number of vesicles and the number of directed runs after MT depolymerization. We cannot discern if these changes are due to the gross alteration in parasite morphology that occurs after oryzalin treatment or if MTs play a minor role in vesicle movement. These alternative possibilities have been stated in the results sections (line 207-208).*

Fig 3B: The Au should perform co-localization studies between the fragmented Rab6 compartment and Golgi markers (notably, a marker for the trans-Golgi as the endosomal-like compartments are tightly associated with the trans-Golgi in T. gondii) to verify that the effect they observe is not an indirect consequence of a loss in Golgi structure/integrity upon CD treatment. One may envision that these Golgi fragments may not be able to functionally interact with the actin/myosin machinery and thus remain static.

*We have performed co-localization experiments between EmGFP-Rab6 and anti-SORTLR, a marker for the trans-Golgi network in both RH parasites and TgMyoF-AID parasites treated with ethanol and IAA (Fig. 1C and S5, respectively). EmGFP-Rab6 and SORTLR compartments are closely aligned in both parasite lines. Upon TgMyoF depletion, some of the fragments of these compartments remain in close proximity while others are dispersed from one another throughout the parasite cytosol.*

*We agree with the reviewer that we cannot conclude that TgMyoF interacts directly with all the organelles affected by loss of TgMyoF. Some of these phenotypes could be indirect effects. We have now modified our conclusions to communicate this point more clearly. Our discussion now reads “This study demonstrates that loss of TgMyoF alters the dynamics, positioning, and movement of a wide array of organelles in the endomembrane pathway in T. gondii. Future studies will be required to elucidate the mechanistic underpinnings of these phenotypes. It will be important to determine if TgMyoF interacts directly with some or all of these organelles or if some of these phenotypes are downstream consequences of disruption to intracellular trafficking caused by the loss of TgMyoF” (Line 505-510).*

Although not mandatory (since the depletion of TgMyoF supports a role for the actin cytoskeleton in Rab6 vesicle movement), the Au may consider to use actin KO parasites to confirm the data they obtain using CD treatment.

*Due to time constraints and scheduling limitations of lab personal due to covid-19, we were unable to complete this experiment.*

Fig5: The Au should take advantage of the rapid inducible AID system to improve the characterization of the PGC defects they described upon TgMyoF depletion. In particular, is the dispersion of the studied endosomal compartments (Rab5/Rab7 and Rab6) observed at early time points of TgMyoF depletion (2h, 3h, 4h) and most importantly, is this phenomenon concomitant or independent of (Cis-/Trans-) Golgi fragmentation.
This could be even achieved by live imaging after recording parasites in G1 phase every 30 min during 30-60s for a duration of 4-6 hours with IAA treatment.

*To address this question, we performed a time course experiment with parasites expressing EmGFP-Rab6 and Grasp-mCherryFP. Parasites were grown for 18 hours without drug and then treated with IAA and fixed every 30 minutes for 7 hours. The number of Golgi per parasite, and number of parasites containing fragmented and distributed Rab6 compartment, were quantified as a function of time. The fragmentation of these organelles occurs concurrently, if anything the Rab6 compartment fragmentation occurs slightly before the Golgi. This data has been added to figure 8.*

*While this experiment was informative it does not fully settle the question of whether TgMyoF interacts directly with both or either of these compartments. In the future, it will be important to elucidate how TgMyoF controls the dynamics of a large number of subcellular compartments and determine if and how this motor directly associates with the membrane of each of these compartments. We have modulated our discussion lines 505-510 to reflect this outstanding uncertainty.*

Fig 8: If TgMyoF is involved in vesicle transport from and at the Trans-Golgi /ELC, alteration of the Golgi structure/function is expected. Could the Au provide electron microscopy images to further detail the structure of the Golgi (they could also document the other organelles, such as the ER and the rhoptries) in TgMyoF-mAID parasites treated with IAA for 4hours and 15hours?

*We thank the reviewer for this suggestion, we are extremely interested in defining the ultrastructure of the organelles in the absence of TgMyoF. Unfortunately, due to time constraints and access to core facilities due to COVID-19, we were unable to perform the EM experiments. We hope to answer this question in a future manuscript.*

Reviewer #2: I have two major issues. First, it was surprising that ER tubule dynamics were not examined in the presence of actin and microtubule inhibitors. Even if this was done previously, I think it’s critical to repeat and reproduce these results. Second, if it’s possible, I would suggest supplementing the figures here with additional images acquired using super resolution microscopy.

*We have now performed imaging of the ER tubule movement in parasites treated with cytochalasinD and oryzalin and included this data in figure 7 and Video 4. A description of this data can be found in the results section (line 297-302). CD treatment results in dramatic inhibition of ER movements and a contraction of the ER tubule network. Oryzalin treatment dramatically perturbs the polarized parasite organization, however ER tubules are still visible through the cytosol of these parasite “masses” and fission and fusion of tubules is still visible suggesting that microtubules are not required for these cellular processes.*

*We would like to obtain super-resolution images of these organelles; however, we do not have access to an OMX structured illumination instrument. Our department has an abberior STED instrument and we have begun the process of obtaining images with this instrument. We are having a number of technical issues which we were unable to resolve in the time frame of this re-submission. We hope to address this in a future manuscript.*

Reviewer #3: Figure 1B-C. The purpose of the experiments in Figure 1 is to establish the localization of Rab6, and the authors conclude that Rab6 localizes to a different compartment to what was previously reported in the literature. The authors examine Rab6 localization by transiently expressing a fluorescent protein-fused Rab6 from a non-native promoter. Transient expression, the use of a large fusion protein, and a non-native promoter all raise concerns about potential artefacts in the observed localization. The authors should test whether a natively-tagged Rab6 protein exhibits a similar localization. e.g. introduce a (smaller) tag into the native locus of Rab6 using established genome editing approaches.

*We agree with the concern of the reviewer and have made numerous attempts to use CrispR-Cas9 to modify the endogenous Rab6 locus to add an N-terminal small epitope V5 tag. Unfortunately, these efforts have not been successful and our repeated attempts have led to the delayed resubmission of our manuscript. We are unable to directly address this concern at this time.*

Figure 5 (and Figures 7-9). The authors should include parental/wild type controls to determine whether the addition of IAA in the absence of TgMyoF depletion influences positioning and appearance of the various post-Golgi compartments that they analyze. I appreciate that it would be considerable work to repeat all the quantificaitons, so perhaps some key controls could be included (e.g. examining the positioning and number of Rab6 compartments in Figure 5, and the positioning/number of Golgi/rhoptries in Figures 8 and 9), with other experiments repeated (and quantified) only if these initial ones reveal concerns about the effects of IAA on processes in the endomembrane system.

*We have completed three control experiments to address this concern. We treated Tir1 parental parasites with ethanol or IAA for 15-18 hours and then quantified the number of apicoplasts/parasite (Fig. S3), the number of Golgi/parasite (Fig. S4) and number of parasites with either intact/apical or fragmented/distributed rab6 compartments (Fig. S4). No differences were observed between ethanol or IAA treated Tir1-parental parasites.*

**Part III – Minor Issues: Editorial and Data Presentation Modifications**
Please use this section for editorial suggestions as well as relatively minor modifications of existing data that would enhance clarity.

Reviewer #1: Fig 2: How the directed nature of the Rab6 vesicle motions has been defined? The methodology should be mentioned within the main text.

*This definition has been added to the results section lines 173-177 and the methods sections lines 660-666.*

Fig 2D (mentioned in the text) is not part of Fig2?
*We have corrected this error.*

-Video 1 shows Rab6-positive vesicles emerging from the Golgi/ELC area (anterograde transport). However, video 2 also shows Rab6(+) vesicle fusion at the Golgi (may be retrograde transport). In mammalian cells, Rab6 is involved in retrograde transport between the Golgi and the ER but also in targeting cargos from the Golgi to the plasma membrane. What is the opinion of the Au on the possible functions of Rab6 in *T. gondii* based on their observations. This should be more clearly discussed in the discussion chapter of the manuscript.

*As we observed previously with dense granules (Heaslip et al 2016; MBoC), the Rab6 vesicles exhibit bidirectional movement towards the apical and basal ends of the parasite. Moreover, individual vesicles frequently change their direction of movement. The vesicle movements are extremely fast which limits us to 1-2 minutes of imaging at a high frame rate. Thus, we cannot track the movement of vesicles “long term”, and we cannot discern their ultimate destination, and unless we observe them budding from the Rab6 compartment, their origin. Although this work provides insight into how these vesicles are transported, we do not think that tracking the movements of these vesicles for short periods of time necessarily gives us insight into what their ultimate destination might be, or the biological role of the Rab proteins on their surface. Elucidating the function of the Rab’s in T. gondii, as well as defining the “pathway” taken by individual cargo proteins before secretion will require an alternative approach and we hope to address these questions in a future publication.*

*Having said that, the reviewer’s point is well taken and we have now added a more detailed description of the Rab6 vesicle movements to the results section (line 173-176) and added a new supplemental figure (Fig. S2) highlighting Rab6 and Rop1 the vesicle movements. In addition, we have added a section to the discussion where we describe the limitations of this data as outlined above (line 451-456).*

-Video 2 nicely shows bi-directional trajectories of Rab6-positive vesicles along the parasite cortex and numerous vesicles at the basal pole of the parasite. May this reflect an activity for Rab6 in recycling? This activity may regulate rhoptry and microneme compartment homeostasis?

*As outlined above, we do not think any conclusions can be made about the role of Rab6 in recycling of secreted proteins, or rhoptry/microneme homeostasis, from visualizing vesicle motions. Future work is required in order to make this determination.*

Fig 3A:
-The Au should add a video (as snapshot images) showing the tracking (trajectory shown) of the Rab6-positive vesicles and the corresponding kymographs. The images in Figure 3A present a dynamic Golgi-like structure (rather than single moving Rab6(+) vesicles) in which it is difficult to assess what has been tracked.

*Time lapse imaging panels have now been added to figure 3 (parts C, F and I; for control, CD- and oryzalin-treated respectively)*

Fig 5B: The number of Rab6-positive vesicles increases in TgMyoF depleted parasites whereas it decreases in presence of cytochalasin D. How do the Au reconciliate this two findings?

*In CD treated parasites there is a decrease in the number of Rab6 vesicles which are typically smaller than 7x7 pixels or ~200µm^2^. However, when we treat with CD or knockdown TgMyoF we observe an increase in Rab6 positive “objects” that are greater than 200 µm^2^. We conclude the while the number of vesicles is decreased, there is an increase in the number of large Rab6 objects which we interpret as a fragmentation of the apical Rab6 compartment. For clarity, additional details have been added to the materials and methods (line 675-678) and the results sections (line 183-186). The results section now reads “First, instead of a single Rab6 compartment at the parasites apical end, we observe multiple large Rab6 structures throughout the parasite cytosol, including the basal end, suggesting that in the absence of F-actin the Rab6 compartment becomes fragmented (Fig. 3D)”*. When we analyze the number of “large” Rab6 compartments we see an increase after both CD treatment and TgMyoF knockdown.

Fig 6: How the Au explain that the velocity of Rab6-positive vesicles is not affected but only the frequency of directed runs? Which mechanisms regulate their motion in absence of TgMyoF? For instance, a transition from directed to diffusive motion should be correlated with changes in vesicle velocity.

*In our analysis, vesicles are only tracked when they are moving in a directed manner. Once the vesicle becomes stationary or exhibits diffusive movement the tracking is terminated. In our experience vesicles will remain diffusive or stationary for longer periods of time compared to the length of time they exhibit directed motion, therefore, including these types of motions in the run-length and velocity measurements artificially lowers these values.*

*We do not know the mechanisms underlying directed vesicle motion in the TgMyoF knockout parasites and we do not know if a small amount of TgMyoF (undetectable by western blot) is still present in some parasites. In other systems, estimates on the number of motors that are found on the surface of a vesicle range from low single digits to <50 (Nelson et al 2014; PNAS; PMID: 25201964) so presumably a small residual pool of TgMyoF may be enough to drive some vesicle movement. Having said that, we don’t know if TgMyoF in fact binds to the surface of membranous cargo, or drives vesicle movement by another mechanism. Without knowing TgMyoF’s mechanism of action it is difficult to discern why motile vesicles from TgMyoF depleted parasites move with similar velocities compared to vesicles from wildtype cells.*

Fig9: The Au wrote in the discussion:
“Our data suggests that TgMyoF is required for movement of immature rhoptries to the apical tip but is not required for TgARO1-dependent anchoring once the organelles have reached their destination.”

Are ROP1 dynamic vesicles co-localizing with any of the endosomal markers used in the study and known to be co-localized with pro-rhoptry proteins? The movie (video 5) shows clear ROP1-positive vesicle transport from the Golgi/ELC area to the apical end of the parasite, likely the mature anchored rhoptries. Some vesicles seem also to bud out the rhoptries? Supporting a role for this transport, in TgMyoF parasites, two almost static ROP1-positive spots (one near the nucleus and one more apical) are observed. The Au could better describe these observations in the “results” chapter page 9.

*We have now expanded our description of the Rop1 movements in the results section (line 382-385) and included new time lapse insets in supplemental figure 2. We have speculated on the function of these vesicles in the discussion (lines 486-492). Future work will be required to answer the outstanding questions on rhoptry formation, and rhoptry protein trafficking and turnover.*

Discussion:
The Au conclude: “This study demonstrates that TgMyoF controls the dynamics, positioning, and movement of a wide array of organelles in the endomembrane pathway in T. gondii. Future studies will be important to elucidate the mechanism by which this single molecular motor controls the movement of such a wide array of membranous cargos.”
The Au should modulate their conclusions as they demonstrated that TgMyoF controls the movement of Rab6-positive vesicles and ROP1-positive vesicles and that TgMyoF depletion leads to a mis-localization of the Rab5/7 compartment, which could be an indirect consequence of the role of TgMyoF in maintaining Golgi function and integrity during parasite replication.

*We have tempered our discussion to this effect, which now reads “This study demonstrates that loss of TgMyoF alters the dynamics, positioning, and movement of a wide array of organelles in the endomembrane pathway in T. gondii. Future studies will be required to elucidate the mechanistic underpinnings of these phenotypes. It will be important to determine if TgMyoF interacts with some or all of these organelles or if some of these phenotypes are downstream consequences of disruption to intracellular trafficking caused by the loss of TgMyoF.” (Lines 505-510)*

Reviewer #2: 1. Figure 1B it is not clear whether or not there is overlap between RAB5 and Golgi marker.
2. Syntaxin 6 may be localized to the IMC and not the plasma membrane

*We have changed this description from “plasma membrane” to “parasite periphery” (line 149)*

3. Figure 2D is referred to in text but is missing.

*This error has been corrected.*

4. It was unclear to me how organelle rearrangements noted in the manuscript (e.g. line 158) is not due to points of interest moving in and out of the focal plane? Perhaps the authors used a confocal imaging system but this wasn’t clear from the Methods section.

*The majority of our live cell imaging was done using the DeltaVision Elite epifluorescence microscope where we image a single Z-plane. While we have performed imaging of Z-stacks, this slows down our imaging frame rate and makes it more difficult to track vesicle movements. Since we observe changes in organelle shape while the organelle is visible and in focus at the focal plane we do not think that the changes in organelle shape are due to movements of the organelle in the Z-direction.*

5. I am not sure how but Figure 7 would benefit from quantification.

*We agree with the reviewer but have not yet been able to develop a method to quantitate ER movements. We have now added line scans to figure 7 to help with the interpretation of this data.*

6. Line 272 should be 8F and 8G

*We have corrected this error.*

7. Line 279: I think that this is a little strong of a conclusion because it's also possible that centrosome positioning is important for Golgi morphology

*We agree with the reviewer and have added this statement to the discussion (line 429-433). The point we were attempting to make on lines 350-353, was that there did not appear to be a block in the cell division cycle after centrosome duplication and Golgi division but that the Golgi appear to fragment in interphase parasites. We have modified this sentence to read “**Thus, we conclude that TgMyoF and actin are important for controlling both Golgi number and apical positioning in interphase parasites and these phenotypes are not due to a block in the cell division cycle after centrosome duplication and Golgi division, the earliest events in the parasite’s cell division cycle.”*

Reviewer #3: Lines 100-101. “*T. gondii* has a single actin gene (TgAct1) that has 83% similarity with chicken skeletal actin (40).” This is an odd comparison to make. Why chicken actin and not that from other animals, or other eukaryotes?

*We have changed this sentence to read “T. gondii has a single actin gene (TgAct1) that has 83% similarity with mammalian β and γ actin isoforms” – the isoforms referenced in the original Dobrowolski et al 1997 paper.*

Line 168-169. “the main Rab6(+) compartment lost its apical localization and became fragmented and distributed throughout the parasite cytosol”. How was the “main Rab6+ compartment” identified? In Figure 3B and Video 2, there seems to be a Rab6+ structure apical to the nucleus.

*The reviewer is correct – there is often a Rab6+ structure at the apical end of the nucleus after CD treatment or TgMyoF depletion. However, there are additional Rab6+ structures distributed throughout the parasite cytosol at the lateral or basal sides of the nucleus. Therefore, we have reworded this description to better reflect this. The sentence now reads* “First, instead of a single Rab6 compartment at the parasites apical end, we observe multiple large Rab6 structures throughout the parasite cytosol, including the basal end, suggesting that in the absence of F-actin the Rab6 compartment becomes fragmented (Fig. 3D).”

Line 175. “directed runs”. The definition of this wasn’t clear to me. Movement of vesicles in a directed manner? And if so, how was directed movement distinguished from non-directed movement? Likewise, the meaning of “run frequency” in the graph in Figure 3D is unclear. The authors should clarify this. The same point applies to the data in Figure 6 and Figure 9E.

*For clarity we have added descriptions of our analysis in the results section (lines 172-176) and defined both directed run and run frequency more clearly. We added additional details of our analysis to the methods section (lines 662-667).*

Figure 3D legend (Line 865). “Error bars indicated mean and SEM”. The mean and SEM of the 3 independent experiments or of the combined observations from all the experiments. I presume the former, but the authors should specify this. Similar comment for Figure 5B (Lines 883-884), Figure 6C (Line 897) and Figure 9E-G (Lines 943-944). Strictly speaking for all of these, the error bars show the variation/SEM, not the mean.

*We have updated all our figure legends to include the following statement. “Mean from each independent experiment is indicated with large circles.* *These values were used to calculate the average (horizontal bar), standard error of the mean (error bars), and P value”.*

Figure S1. The PCR screens that were undertaken, and the associated schematic images, need a clearer description. What are the expected sizes of the various PCR bands? What is the ‘yellow’ box depicted in the bottom schematic? The authors should also include a parental control in these screens (i.e. to determine the sizes/presence of bands in an unmodified locus).

*We have now included PCRs from parental control and show the expected product sizes in the figure (now Fig. S3). The construct used to make this parasite line originated from pTKOII-MyoF-EmeraldFP described in Heaslip et al 2016. The yellow box represents a loxP site which was inserted into intron 18 in the original parental construct. This description has been added to the figure legend for clarity.*

Figure S2B. The authors should include a parental control to determine the effects of IAA on apicoplast inheritance. The authors could also consider including other measures to ascertain whether mAID-mediated MyoF knockdown phenocopies the effects of MyoF loss reported in previous studies (e.g. does knockdown of MyoF-mAID cause a defect in parasite proliferation?)

*We have completed three control experiments to address this concern. We treated Tir1 parental parasites with ethanol or IAA for 15-18 hours and then quantified the number of apicoplasts/parasite (Fig. S3), the number of Golgi/parasite (Fig. S4) and number of parasites with either intact/apical or fragmented-distributed rab6 compartments (Fig. S4). No differences have been observed between ethanol or IAA treated Tir1-parental parasites.*

Figure 8H. How long were parasites incubated in IAA in these experiments? Should specify this in the figure legend or main text.

*This information has been added to each figure legend*

Lines 346-348. “We have identified Rab6 as a new marker of the syntaxin 6 compartment, a protein that plays a role in retrograde trafficking from the Rab5a/Rab7 compartments to the Golgi”. I think the authors mean that syntaxin 6 plays a role in retrograde trafficking, whereas the text suggests Rab6 does (or that the “syntaxin 6 compartment” is a protein, which doesn’t make sense). This needs to be re-written for clarity.
*This sentence has been re-written to read “Syntaxin6 is a protein previously shown to play a role in retrograde trafficking from the Rab5a/Rab7 compartments to the Golgi”*

Lines 354-355. “This data is consistent with a recent report demonstrating that Rab11a is found on the surface of dense granules”. I don’t understand the meaning here. Why would Rab6 not co-localizing with dense granules be consistent with Rab11a localizing with dense granules. The authors should be clearer here.
*We have removed this sentence from the manuscript.*

Lines 394-395. “where there is increased accumulation of rhoptries throughout the parasite cytosol”. I’m not sure it is right to call the ROP1-GFP-labelled structures observed in the MyoF knockdown parasites rhoptries. The data that the authors present in Figure 9 seem to show that the ROP1-GFP protein localizes in smaller vesicles rather than intact, club-shaped rhoptries. As the authors suggest at the end of the paragraph, these might represent immature rhoptries or vesicles containing rhoptry material. Better to reword to “…where this is increase accumulation of the rhoptry protein marker to vesicles throughout the parasite cytosol” or similar.

*This sentence has been reworded.*

Minor comments on the text and grammar
*All text and grammar errors listed below have now been corrected.*

Throughout: Golgi (uppercase “G”) vs golgi (lowercase “g”). Should be consistent.

Line 79. “Rab6, is thought to localizes to…”- “thought to localize to”

Line 107-108. “including apicoplast inheritance (a non-photosynthetic plastid organelle)”. Apicoplast inheritance is not an organelle … Perhaps: “including in the inheritance of the apicoplast (a non-photosynthetic plastid organelle)”

Line 185. “referred to subsequently as TgMyoF-AID”. Some of the figures refer to this as “TgMyoF-mAID” (Figure 4 and 6)

Line 191. “Fig. S1B” - should refer to Fig. S2B.

Line 217. “compartment’s” not “compartments”. No full stop before “(Fig. 6A …)”.

Line 271. “twp” – “two”

Line 296. “parasite’s” – no apostrophe

Line 299. “Video 6”. This wasn’t present in my version of the manuscript. Do the authors mean Video 5, which also appears to show moving ROP1 vesicles?

Line 334. “… could be … may be …” - vague. Perhaps just “…is that the Golgi may be comprised of …”

Line 354. “This data …” – “These data …” (plural)

Line 358. “compartments is” – singular or plural? Later in this sentence - “… rearrangement, we observed …” – does the “we” need to start a new sentence?

Line 364. “along BAR domain…” – “along with”?

Line 433. “as indicated in figure 3.” Figure 4, no?
